# Jointly discussing care plans for real-life patients: The potential of a student-led interprofessional team meeting in undergraduate health professions education

**DOI:** 10.1007/s40037-019-00543-6

**Published:** 2019-11-08

**Authors:** Marion van Lierop, Jerôme van Dongen, Miriam Janssen, Hester Smeets, Loes van Bokhoven, Albine Moser

**Affiliations:** 1 Department of Family Medicine, Maastricht University, Maastricht, The Netherlands; 2 Research Centre for Community Care, Zuyd University of Applied Sciences, Heerlen, The Netherlands; 3 CAPHRI School for Public Health and Primary Care, Department of Family Medicine, Maastricht University, Maastricht, The Netherlands; 4 Department of Social Medicine, Maastricht University, Maastricht, The Netherlands; 5 Faculty of Health, Zuyd University of Applied Sciences, Heerlen, The Netherlands; 6 Research Centre for Autonomy and Participation of people with chronic illnesses, Zuyd University of Applied Sciences, Heerlen, The Netherlands

**Keywords:** Interprofessional learning, Interprofessional education, Interprofessional team meeting, Problem based learning

## Abstract

**Background:**

Interprofessional education is promoted as a means of enhancing future collaborative practice in healthcare. We developed a learning activity in which undergraduate medical, nursing and allied healthcare students practice interprofessional collaboration during a student-led interprofessional team meeting.

**Design and delivery:**

During their clinical rotation at a family physician’s practice, each medical student visits a frail elderly patient and prepares a care plan for the patient. At a student-led interprofessional team meeting, medical, nursing and allied healthcare students jointly review these care plans. Subsequently, participating students reflect on their interprofessional collaboration during the team meeting, both collectively and individually. Every 4 weeks, six interprofessional team meetings take place. Each team comprises 9–10 students from various healthcare professions, and meets once. To date an average of 360 medical and 360 nursing and allied healthcare students have participated in this course annually.

**Evaluation:**

Students mostly reported positive experiences, including the opportunity to learn with, from and about other healthcare professions in the course of jointly reviewing care plans, and feeling collectively responsible for the care of the patients involved. Additionally, students reported a better understanding of the contextual factors at hand. The variety of patient cases, diversity of participating health professions, and the course material need improvement.

**Conclusion:**

Students from participating institutions confirmed that attending a student-led interprofessional team meeting had enabled them to learn with, from and about other health professions in an active role. The use of real-life cases and the educational design contributed to the positive outcome of this interprofessional learning activity.

## Background

The World Health Organization (WHO) states that ‘interprofessional education occurs when students from two or more professions learn about, from and with each other to enable effective collaboration and improve health outcomes’ [1]. The WHO indicates in this framework that ‘in both acute and primary care settings, patients report higher levels of satisfaction, better acceptance of care and improved health outcomes following treatment by a collaborative team’ [1]. To date, the curricula of most healthcare training programs in the Netherlands have incorporated interprofessional collaboration (IPC) as a key competency for their graduates [2–4]. Interprofessional education (IPE) is promoted as a means of enhancing IPC in the future [5].

Effective integration of IPE into the curricula of health professions training requires efficient collaboration between the various stakeholders to overcome barriers to IPE, such as logistical challenges, faculty attitudes, a rigid curriculum or differences in assessment requirements [6]. Recommendations to deploy constructivist learning theory to underpin interprofessional learning activities, to stress student-centred learning and to create meaning from the interprofessional learning experience all pose further challenges to IPE [5].

With these issues in mind, in 2015 Maastricht University, Maastricht, the Netherlands and Zuyd University of Applied Sciences, Heerlen, the Netherlands, jointly developed an IPE course for medical, allied healthcare (i.e. physiotherapy, occupational therapy, speech and language therapy) and nursing students. The goals of the IPE course were: (1) to experience the practice of direct collaboration with other future health professionals; (2) to feel collectively responsible for the outcomes of an interprofessional team meeting; and (3) to create an opportunity to reflect on IPC, since reflection is paramount in competency-based medical education, as it steers the learning cycle [7].

At the start of this project, we recognized the dissimilarities in the design of the educational programs at both participating institutions, such as differences in the length of training, intended learning outcomes and target competencies [2–4]. Medical training at Maastricht University involves a 6-year undergraduate program entailing a 3-year Bachelor’s phase and an equally long Master’s phase [8]. Medical training comprises a competency-based program, taking into account the outcomes as laid down in the Dutch Framework for Undergraduate Medical Education [4], which is based on the CanMeds competency framework [9]. This framework states that by graduation, medical students should be able to make an effective contribution to interprofessional teams. The described IPE course is part of Maastricht University students’ clinical rotation in family medicine and social medicine during the Master’s in medicine [8]. Health professions training programs at Zuyd University comprise 4‑year Bachelor’s programs [10], each with its own curriculum and intended learning outcomes based on nationwide frameworks [2, 3]. Zuyd University has built a framework of interprofessional competences, known as ‘interprofessional building blocks’, based on existing competence models. It comprises their five key adopted competencies for IPC, which are implemented in all allied healthcare and nursing students’ training programs [11]. These competencies are: (1) knowing and understanding each other’s competences; (2) working with interprofessional care plans; (3) problem-solving in interprofessional teams; (4) appropriate interprofessional referral; and (5) evaluation of interprofessional teamwork. At Zuyd University, the described IPE course is embedded in the third and fourth year of allied healthcare and nursing Bachelor’s programs [10].

Our joint effort resulted in an IPE course including: (1) participation in a student-led interprofessional team meeting; (2) jointly composing a care plan for a frail elderly patient, and (3) subsequent reflection (team and individual) on IPC. We expected that students would gain knowledge about and comfort in working with other healthcare professionals during this innovative IPE course. By now, around 360 medical and 360 allied healthcare and nursing students have participated in this IPE course every year since it started in January 2015.

## Design and delivery

An interprofessional team of faculty of both universities developed the course. The intended learning outcomes of the IPE course were based both on the interprofessional building blocks (Zuyd University) [11] and the outcomes of the Dutch Framework for Undergraduate Medical Education (Maastricht University) [4]. This means that all students should be able to learn how to make an effective contribution to an interprofessional team in the field of patient care, as well as how to develop a care plan for a patient in consultation with other healthcare professionals. As the ageing society is leading to more patients with chronic illnesses who are in need of care, often from multiple healthcare professionals [12], we agreed to focus on care for the frail elderly. Frail elderly patients are professionally relevant to all medical, allied healthcare and nursing students.

**Fig. 1:**
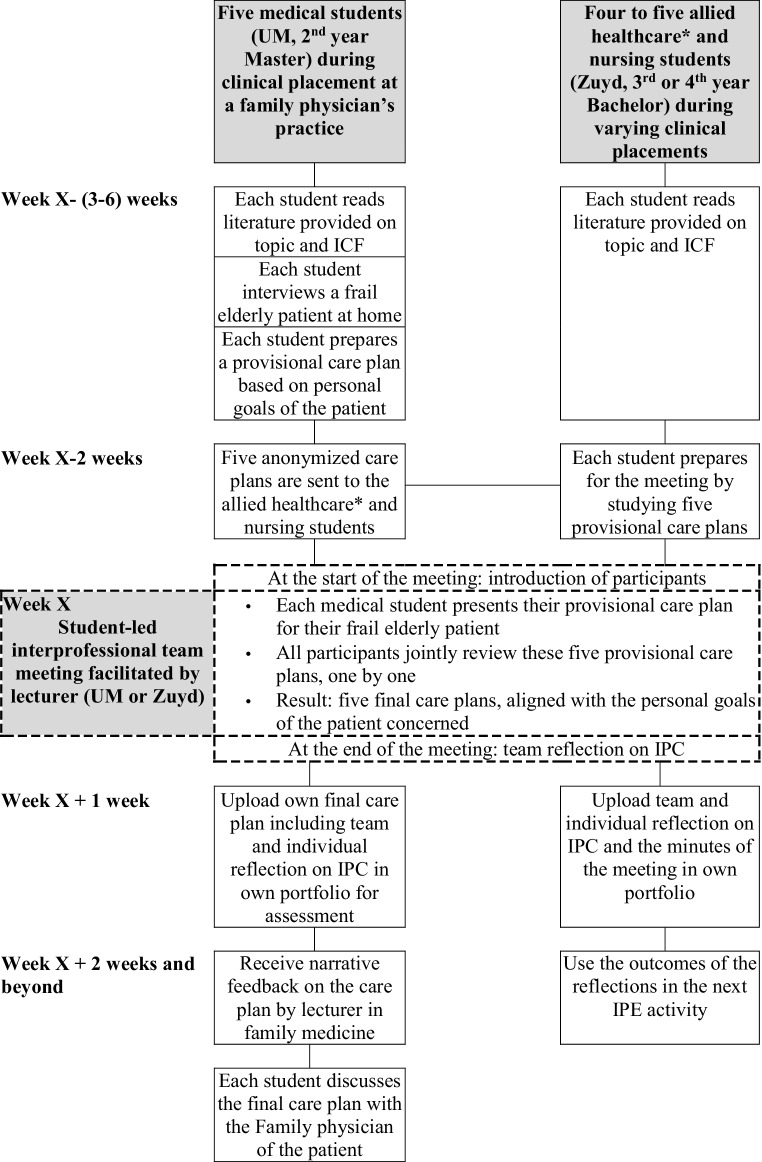
Road map to a student-led interprofessional team meeting where participants jointly discuss care plans for frail elderly patients. *UM* Maastricht University; *ICF* World Health Organization’s International Classification of Functioning, Disability and Health; *IPC* interprofessional collaboration; *IPE* interprofessional education. * physiotherapy, occupational therapy, speech and language therapy students

The design of this IPE course, i.e. jointly discussing care plans for frail elderly patients during a student-led interprofessional team meeting, was based on the key principles of problem-based learning [13]. Firstly, it is constructive, because students activate prior knowledge, elaborate on what they have learned and by focusing on real-life cases they trigger deep learning. Secondly, it is collaborative, because students from differing health professions work together on a care plan, whereby they get a view of the perspective of other health professions involved in caring for frail elderly patients. Thirdly, it is contextual, because students deploy real-life instead of made-up patient cases for the construction of a care plan. Lastly, learning is self-directed as students plan, monitor, evaluate and reflect on their own learning. We developed a road map, tailored to the health professions of participating students, in which we describe the steps that should be taken to be optimally prepared for participation in the student-led interprofessional team meeting (Fig. 1). Students are also provided with literature on how to design a care plan [14] and how to use the WHO’s International Classification of Functioning, Disability and Health (ICF), promoted as the common language for health professionals [15].

## Road map

### *Interprofessional teams*

Every 4 weeks six interprofessional teams are formed, each comprising 9–10 students from different health professions (5 medical students and 4–5 allied healthcare and nursing students). We attempt to include at least one student from the department of physiotherapy, occupational therapy, speech and language therapy and nursing in each team. For each team, one interprofessional team meeting is scheduled.

### Medical students

Firstly, the medical student visits a frail elderly patient at home, takes a medical history, and asks the patient about their personal goals. The medical student then draws up a provisional care plan (based on the ICF), and sends the anonymized provisional care plan to the allied healthcare and nursing students of their interprofessional team 2 weeks before the student-led interprofessional team meeting takes place. We assigned this task to the medical students for pragmatic, mainly logistic, reasons. The IPE course is scheduled during their rotation in family medicine and social medicine. In this way we can guarantee that medical students can actually visit a frail elderly patient at home and have sufficient time to prepare for the interprofessional team meeting. After the meeting, each medical student finalizes the care plan for their frail elderly patient, based on the outcomes of the meeting as laid down in the minutes of the meeting. They add the team reflection and, together with an individual reflection on IPC (Tab. 1), upload the final care plan to their portfolio for assessment and narrative feedback from a lecturer in family medicine. The medical student should also discuss the final care plan with the patient’s family physician afterwards with the intention of implementing it.

**Table 1:** Example of a team and an individual reflection on IPC

*Part of a team reflection, written by a medical student (interprofessional team meeting Y)*
… ‘All disciplines thought that the meeting was useful. It was useful to hear the other allied healthcare and nursing students explaining their differing visions on things. Their tips and advice has helped us medical students to look at a patient case from a broader perspective and to prevent us from limiting ourselves to treating the symptoms only. Several methods of approaching a number of physical and cognitive problems were discussed. The good thing about it was the fact that a number of approaches were discussed which would perhaps not immediately occur to a physician, but other areas of healthcare would have a solution. The interdisciplinary exchange of views was an enriching experience, which once again made it clear that as a physician you are not only there for the health problems of your patients. You need to be aware that there are many other services and caregivers that you can (and sometimes must) contact, formally or informally, in order to provide good and complete care to your patients.’…
*Part of an individual reflection, written by an allied healthcare student (interprofessional team meeting Z)*
… ‘During the team meeting we worked together on a number of cases from practice. In this, we worked more on a practical level and looked at what each of the different disciplines could do for a client. By doing this, you gain good insight into working with other disciplines. It also gives you an impression of how a team meeting is conducted and the sort of thing that is discussed there. At Zuyd we had already collaborated with other disciplines, but not with other institutions. What I found so interesting was how much interest medical students showed in what the Zuyd students had to say. I hadn’t expected this. My take home message for the future is that other disciplines can help and support you really well. They can make you see things from a different perspective and introduce new possibilities. Further, when I am compiling a treatment plan, I will be able to focus more on finding out what other disciplines would be able to do for the client. Now, the focus is all on occupational therapy and not what others could do for the client. I can continue to reflect on this, and improve my competence in the management and treatment of the client.’ …

### Allied healthcare and nursing students

Allied healthcare students (physiotherapy, occupational therapy, speech and language therapy students) and nursing students study the provisional care plans they receive from the medical students 2 weeks before the interprofessional team meeting takes place. In order to be well prepared for this meeting, we ask them to consider how their own profession might contribute to the realization of the patient’s personal goals. Following the student-led interprofessional team meeting, students upload the team reflection of the interprofessional meeting they participated in, including an additional individual reflection on IPC (Tab. 1) as well as the minutes of the meeting to their own portfolio. This material is then deployed in other IPE activities at Zuyd University.

### *Student-led interprofessional team meeting*

Every 4 weeks, six student-led interprofessional team meetings, each lasting 2.5 h, take place concurrently. Lecturers in family medicine and social medicine (Maastricht University), and allied healthcare and nursing teaching staff (Zuyd University) facilitate these interprofessional team meetings. The role of the facilitator is to request clarification should it be necessary, to correct potentially erroneous proposed solutions concerning the care for the frail elderly patient, and to ensure that the team reflection on IPC takes place at the end of the meeting.

The student-led interprofessional team meeting starts with a short introduction by the facilitator explaining the purpose of the meeting. Each participant is then given the opportunity to introduce themselves and provide the interprofessional team members with information on their future role in patient care. In order to break traditional patterns [5], one of the allied healthcare or nursing students is requested to chair the interprofessional team meeting and a second one to take the minutes. Next, each medical student presents their frail elderly patient and their provisional care plan. In this way, five care plans are reviewed during the interprofessional team meeting. Participants then jointly review the care plans and arrive at a final proposal for the best possible care for the patient. At the end of the meeting, 30 min are allocated for team reflection on IPC, covering such items as atmosphere, interaction, leadership, what students have learned about other participating health professions, and whether collaboration has been conducted respectfully. Afterwards, the minutes of the meeting, including the team reflection, are sent to all participants.

## Evaluation

In mid-2016, we conducted four focus group meetings in order to evaluate the IPE course. One of the focus groups comprised 5 medical students (Maastricht University) and the remaining three groups comprised Zuyd University students, i.e. a total of 5 physiotherapy students, 6 occupational therapy students, 4 speech and language therapy students and 4 nursing students. We assumed that the interaction between participants during the focus group meetings could lead to more in-depth insights [16].

At the start of each focus group meeting, the facilitator (HS or JvD) asked students to describe three positive and three negative experiences of the student-led interprofessional team meeting, as a sensitizer for the topic. Students then elaborated on their remarks in a plenary discussion on learning benefits. Follow-up questions were used to gain more in-depth information on learning benefits. The focus group meetings were audio-taped and transcribed verbatim and analyzed by means of inductive conventional content analysis by HS, JvD, MJ and MvL [17].

Students’ experiences with this IPE course were mainly positive. Positive experiences included the opportunities to (1) learn more about the work of other health professions; (2) get a more extensive perspective on the patient’s problems and personal goals, enabling them to better understand contextual factors and stimulating them to employ a more patient-centred view; and (3) to learn through real-life frail elderly patients, which gave them a feeling of responsibility for the final care plan for the patient. The safe learning environment during the interprofessional team meeting, including mutual respect for each other’s expertise, and the opportunity to eliminate stereotypical prejudices about other health professions was also valued.

Participants also noted points for improvement. These included: (1) lack of variety of patient cases, as all patients were frail elderly people (2) lack of diversity in participating health professions, since not every allied healthcare profession was always represented at the interprofessional team meetings; (3) the course material and road map could have been more clear and concise; (4) in order to save time during the introduction phase of meeting, there was a need to become familiar with the roles of other participating disciplines before the meeting; (5) the IPE course could be better integrated into and aligned with the various curricula.

The focus group meetings mentioned were part of the regular program evaluation of this IPE course. In accordance with the Declaration of Helsinki, students received information about the evaluation, anonymity and confidentiality. They consented to participate in a focus group meeting on a voluntary basis and participation was of no influence on the outcomes of the IPE course for the individual student concerned.

## Discussion

In this IPE course, based on the principles of problem-based learning [13], medical, allied healthcare and nursing students from different higher education institutions are given the opportunity to learn from and about other health professions. During a student-led interprofessional team meeting they jointly discuss care plans for frail elderly patients living at home. Afterwards they reflect on interprofessional collaboration.

Participating students appreciated this IPE course mainly because of the use of real-life cases, which is in line with earlier findings from Gilligan and colleagues [18], who found that IPE experiences that involved genuine engagement and opportunities to interact were valued most. Visser and colleagues [19] too, found that ‘active participation and more self-guided learning of students in the IPE activity led to more satisfaction and improvement of the perceptions of other professions’, which resembles our findings.

Some suggestions for improvement have already been implemented: (1) to improve the diversity of participating disciplines, students from arts therapy and secondary vocational nursing education now participate in this IPE course; (2) we improved the course material and road map by adding a timeline and making it more concise and focused on what the role of each participating health professions student should be during the IPE course; and (3) we developed short video clips in which one student from each participating healthcare profession tells about the possible contribution of their future discipline to the care of frail elderly patients. In future evaluation we will assess whether the described measures are indeed improvements.

We are currently working on the other suggestions for improvement. Firstly, to improve the variety of cases, we are considering to also include patients of all ages with complex multimorbidity. Secondly, we are discussing the possibility to also allow one or more of the participating allied healthcare or nursing students to propose a provisional care plan for a real-life patient to be discussed during the interprofessional team meeting. Thirdly, we look forward to including social work students in the near future to further enhance the diversity of participating students. Fourthly, the integration of this IPE course is an ongoing process in which the development of longitudinal interprofessional curricula at both universities could be a means to facilitate alignment. Lastly, we are aware that only the medical students conduct the assessment and that, based on this information, allied healthcare students and nursing students suggest a treatment plan. It seems a great IPE opportunity for all team members to meet the same community-based patient, maybe jointly. However, we are confined by the reality of logistics, working with two separate higher education institutes. Allied healthcare students and nursing students have different practice placements and different time schedules than the medical students. Next to this, we are also concerned that several assessments could be a great burden for the frail elderly patients involved.

## Conclusion

The goals of our IPE course are for the students to experience the practice of interprofessional collaboration, jointly composing a care plan for a frail elderly patient, and to reflect on IPC. We conclude that a student-led interprofessional team meeting in undergraduate health professions education has the potential to practice interprofessional collaboration as it will provide many students with the opportunity to learn with, from and about other health professions in an active way. The use of real-life cases and the educational design contributes to the positive outcome of this IPE activity, giving students the feeling of collectively being responsible for the suggested care for the patient concerned. Paramount in the design, development and implementation of the IPE course is the close collaboration between staff from both participating higher education institutions including their willingness to overcome barriers to IPE.

## References

[1] World Health Organization (2010). Framework for action on interprofessional education and collaborative practice.

[2] Toekomstbestendige beroepen in de verpleging en verzorging: rapport stuurgroep over de beroepsprofielen en de overgangsregeling. 2015. https://www.nfu.nl/img/pdf/Rapport_toekomstbestendige-beroepen-in-de-verpleging-en-verzorging.pdf. Accessed 29 Mar 2019.

[3] KNGF. Beroepsprofielen fysiotherapeut. https://www.kngf.nl/vakgebied/vakinhoud/beroepsprofielen.html. Accessed 29 Mar 2019.

[4] Laan R, Leunissen R, Van Herwaarden C (2010). The 2009 framework for undergraduate medical education in the Netherlands. TMO.

[5] Thistlethwaite J (2012). Interprofessional education: a review of context, learning and the research agenda. Med Educ.

[6] Lawlis TR, Anson J, Greenfield D (2014). Barriers and enablers that influence sustainable interprofessional education: a literature review. J Interprof Care.

[7] Carraccio CL, Englander R (2013). From Flexner to competencies: reflections on a decade and the journey ahead. Acad Med.

[8] Maastricht University. Master geneeskunde. https://www.maastrichtuniversity.nl/nl/onderwijs/master/master-geneeskunde. Accessed 29 Mar 2019.

[9] Royal College. Welcome to CanMEDS interactive. http://canmeds.royalcollege.ca/en/about. Accessed 29 Mar 2019.

[10] Zuyd University of Applied Sciences. Opleidingen. https://www.zuyd.nl/opleidingen. Accessed 29 Mar 2019.

[11] Faculty working group Interprofessional Education (IPE). Interprofessional competence model and interprofessional building blocks. http://docplayer.nl/22387139-Interprofessional-competence-model-and-interprofessional-building-blocks.html. Accessed 29 Mar 2019.

[12] World Health Organization. The world health report 2008—primary health care (now more than ever). https://www.who.int/whr/2008/en/. Accessed 29 Mar 2019.

[13] Dolmans DH, De Grave W, Wolfhagen IH, Van der Vleuten CP (2005). Problem-based learning: future challenges for educational practice and research. Med Educ.

[14] Zorginstituut Nederland. Raamwerk individueel zorgplan 2012. https://www.zorginzicht.nl/kennisbank/Paginas/Raamwerk-Individueel-Zorgplan.aspx. Accessed 29 Mar 2019.

[15] World Health Organization. International classification of functioning, disability and health (ICF) 2001. http://www.who.int/classifications/icf/en/. Accessed 29 Mar 2019.

[16] Krueger R, Casey M (2015). Focus groups: a practical guide for applied research.

[17] Hsieh HF, Shannon SE (2005). Three approaches to qualitative content analysis. Qual Health Res.

[18] Gilligan C, Outram S, Levett-Jones T (2014). Recommendations from recent graduates in medicine, nursing and pharmacy on improving interprofessional education in university programs: a qualitative study. BMC Med Educ.

[19] Visser CLF, Ket JCF, Croiset G, Kusurkar RA (2017). Perceptions of residents, medical and nursing students about Interprofessional education: a systematic review of the quantitative and qualitative literature. BMC Med Educ.

